# Micronized Palmitoylethanolamide Reduces the Symptoms of Neuropathic Pain in Diabetic Patients

**DOI:** 10.1155/2014/849623

**Published:** 2014-04-02

**Authors:** Chiara Schifilliti, Lelio Cucinotta, Viviana Fedele, Carmela Ingegnosi, Salvatore Luca, Carmelo Leotta

**Affiliations:** Associazione MOV.I.S, Onlus di Giarre, Via Teatro 81, Giarre, 95014 Catania, Italy

## Abstract

The present study evaluated the effectiveness of micronized palmitoylethanolamide (PEA-m) treatment in reducing the painful symptoms experienced by diabetic patients with peripheral neuropathy. PEA-m, a fatty acid amide of the N-acylethanolamine family, was administered (300 mg twice daily) to 30 diabetic patients suffering from painful diabetic neuropathy. Before treatment start, after 30 and 60 days the following parameters were assessed: painful symptoms of diabetic peripheral neuropathy using the Michigan Neuropathy Screening instrument; intensity of symptoms characteristic of diabetic neuropathic pain by the Total Symptom Score; and intensity of different subcategories of neuropathic pain by the Neuropathic Pain Symptoms Inventory. Hematological and blood chemistry tests to evaluate metabolic control and safety were also performed. Statistical analysis (ANOVA) indicated a highly significant reduction in pain severity (*P* < 0.0001) and related symptoms (*P* < 0.0001) evaluated by Michigan Neuropathy Screening instrument, Total Symptom Score, and Neuropathic Pain Symptoms Inventory. Hematological and urine analyses did not reveal any alterations associated with PEA-m treatment, and no serious adverse events were reported. These results suggest that PEA-m could be considered as a promising and well-tolerated new treatment for symptomatology experienced by diabetic patients suffering from peripheral neuropathy.

## 1. Introduction

Neuropathic pain is a frequent and serious complication of diabetic neuropathy which markedly impacts a patient's quality of life and, in particular, sleep and daily activities [1]. More than 50% of all diabetic patients suffer peripheral neuropathy, while one-half of patients affected by peripheral neuropathy are diabetic. Distal symmetrical sensorimotor polyneuropathy is the most commonly reported form of diabetic neuropathy. Clinical manifestations are characterized by painful symptoms, often associated with nocturnal exacerbations, described as deep, sharp, and stinging pain, and burning with hyperalgesia/allodynia, often accompanied by a progressive decrease in sensitivity [2, 3]. The pathophysiological mechanisms underlying painful diabetic neuropathy remain largely unknown; recent evidence suggests the involvement of multiple and complex factors, apart from glycemic control and disease duration, such as the reduction in K^+^ channel activity that may have a role in regulation of primary sensory neuron excitability and pain sensitivity [4].

The inability to prevent the onset of neuropathy in diabetic patients, even with good glycemic control, suggests that a dysregulation in the secretion of proinflammatory mediators may underlie the pathogenesis of neuropathic painful symptoms. In fact these mediators, independently of the initial metabolic stimulus and because of their pleiotropic effects, affect glial and neuronal cell homeostasis in the central and peripheral nervous systems. They are secreted by resident tissue cells or infiltrating cells, such as macrophages, mast cells, and lymphocytes [5]. Considering the effects of these mediators on the structural and functional properties of nerve fibers, interventions that modulate their release represent a possible therapeutic approach to alleviating the painful symptoms of diabetic peripheral neuropathy. Numerous studies have demonstrated that palmitoylethanolamide (PEA), a fatty acid amide of the N-acylethanolamine family, is capable of exerting important analgesic, anti-inflammatory, and neuroprotective effects acting on several molecular targets in both the central and peripheral nervous systems as well as in immune cells. PEA has been suggested to act by downregulating mast cell degranulation via an “autacoid local inflammation antagonism” (ALIA) effect [6]. The “entourage effect,” instead, posits that PEA acts by enhancing the anti-inflammatory and antinociceptive effects exerted by anandamide or other endocannabinoid-like molecules. PEA potentiates anandamide action at cannabinoid CB1 and CB2 receptors and/or transient potential vanilloid receptor type 1 channels, while having no appreciable affinity for these receptors [7–10]. However, in one study, transient potential vanilloid receptor type 1-expressing small sensory neurons were reported to be not involved in the development of allodynia in a rat model of diabetic neuropathic pain [11]. Certain anti-inflammatory and antihyperalgesic actions of PEA may also be mediated by a direct activation of peroxisome proliferator-activated receptor alpha (PPAR-*α*) through Ca^2+^-activated K^+^ channels [12–14]. PEA may interact also with other members of the PPAR family to elicit its anti-inflammatory activity [15, 16]. Furthermore, it has been reported that PEA induces de novo synthesis of neurosteroids acting on *γ*-aminobutyric acid A receptors that in turn could contribute to pain perception involving chloride inward flux [17, 18]. The anti-inflammatory and analgesic properties of PEA have been observed also in chronic and/or neuropathic pain. PEA actions are believed to operate via mast cells and microglia, nonneuronal cell populations implicated in the maintenance of neuroimmune homeostasis and in the development of inflammatory processes [19–23]. Administration of PEA in an experimental model of diabetes was effective in relieving neuropathic pain without altering glycemic status [24].

Clinical results obtained in a large number of patients, suffering from neuropathic pain associated with pathologies of various etiology, provide ample support for the anti-inflammatory and analgesic effects of micronized PEA (PEA-m particle sizes 2,0 ÷ 10,0 *μ*m) [25–32]. These findings support the view that PEA-m therapeutic activity is independent of pain etiopathogenesis, but it is rather related to a mechanism having a substrate common (mast cells and microglia) to the various diseases [33]. With this pharmacological and clinical evidence in mind, the present study was designed to assess the effects of PEA-m on painful symptoms in diabetic patients with peripheral neuropathy.

## 2. Materials and Methods

### 2.1. Patients

The patients were selected among the diabetic patients attending the MOV.I.S. Onlus Health Care Center in Giarre (Catania, Italy). The specific inclusion/exclusion criteria are detailed below.

The Health Care Center receives patients diagnosed with various chronic diseases (dysmetabolic, respiratory, etc.) undergoing continuous/periodic monitoring and clinical control by medical specialists. From this population, 30 patients, having confirmed diagnosis of Type II diabetes mellitus and complaining of painful symptoms of peripheral neuropathy, were screened. Patient inclusion was based on the following criteria: diagnosis of Type II diabetes mellitus; satisfactory metabolic compensation; and presence of moderate symptoms of painful diabetic neuropathy as judged by the Michigan Neuropathy Screening Instrument (MNSI, score > 2) [34, 35], verified also by neurological examination and by the Total Symptom Score (TSS) to confirm the presence of characteristic neuropathic symptoms [36]. Excluded patients were those with non-Type II diabetes; noncompensated metabolic syndrome; borderline or severe neuropathic symptoms as indicated by the MNSI score; and peripheral neuropathy due to diseases other than diabetes mellitus. Also excluded were patients judged to be unreliable or noncompliant with respect to treatment.

In accordance with guidelines established for Good Clinical Practice, the study protocol was communicated to the health care managers of the MOV.I.S. Onlus. The study was conducted, according to the ethical principles set out in the Declaration of Helsinki and its revisions. All eligible patients were properly informed about the study and gave their written informed consent to participate.

### 2.2. Study Design

This was an open-label study, in which all patients were treated with PEA-m (Normast 300 mg, 2 tablets daily;* Epitech Group srl, Saccolongo, Italy)* for 60 days. They were allowed to continue with their usual treatment if they had other comorbidities.

### 2.3. Parameters Evaluated

At the time of recruitment, all patients underwent a thorough medical history, clinical and neurological examination, and laboratory tests (hematology, blood chemistry). Particular attention was given to the presence of signs and symptoms of diabetic peripheral neuropathy. For this purpose we made use of the MNSI score (a questionnaire assessing the symptoms of diabetic peripheral neuropathy [35]), which provides a valid and noninvasive measurement of diabetic neuropathy. The TSS was used to assess the intensity and frequency of symptoms such as neuropathic pain, burning, paresthesia, and numbness/lack of sensitivity. Finally, the diverse manifestations of neuropathic pain were analyzed in depth by means of the Neuropathic Pain Symptom Inventory (NPSI). The latter describes the intensity for different categories of pain symptoms and the duration and degree of crisis of paroxysmal pain. The NPSI also allows for grouping of the various pain symptoms into subcategories, such as spontaneous superficial burning pain, deep spontaneous pressing pain, paroxysmal pain, and evoked pain (paresthesia/dysesthesia), that best characterize neuropathic pain. This last analysis provides detailed information about individual pain features and is also very sensitive to quantifying the response to therapy [37, 38]. These evaluations were performed prior to start of therapy (baseline, T0) and again after 30 days (T30) and 60 days (T60) of treatment. One month after treatment end (T90) all patients were subjected to a follow-up visit to assess their general clinical picture, using the TSS and MNSI.

During the treatment period, the assessment of safety and tolerability of PEA was carried out by monitoring the onset and/or reports of adverse events and by hematology and blood chemistry/urine tests performed at baseline and at treatment end.

### 2.4. Statistical Analysis

Data relative to evaluation of pain symptoms were analyzed by Analysis of Variance (ANOVA) for repeated measures. Statistical significance was taken at *P* < 0.05. The McNemar Test was used instead to evaluate the haematological and blood chemistry analyses performed at baseline and at treatment end.

## 3. Results and Discussion

Thirty patients, 15 males and 15 females, between the ages of 53 and 86 (mean 68.3 ± 9.4 years) affected by Type II diabetes (mean time from onset 18.2 ± 9.0 years) and complaining of neuropathic painful symptoms were enrolled in the study between March and November, 2010. Patient medical histories revealed the presence of the following comorbidities: hypertension, 46%; cardiopathies, 10%; and chronic obstructive pulmonary disease, Parkinson's disease, and multiple sclerosis, 3%. The study was completed by 96.7% of the patients (29); one patient withdrew 10 days after the start of treatment, citing excessive sleepiness.

**Figure 1:**
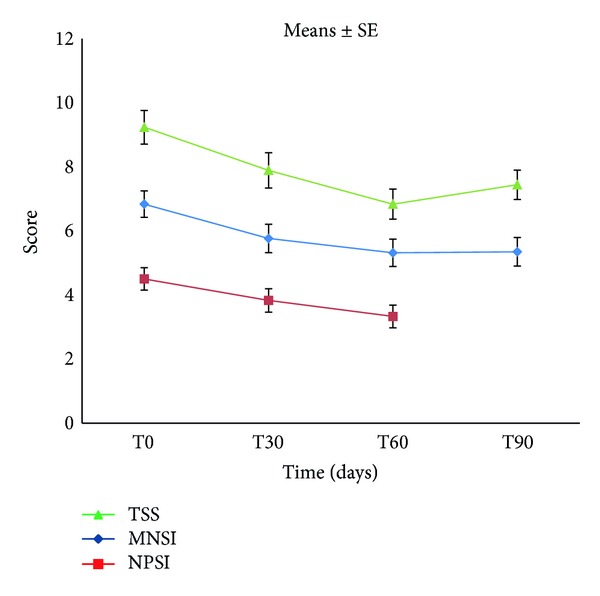
Effect of micronized PEA on diabetic painful neuropathy evaluated by Michigan Neuropathy Screening Instrument (MNSI), Total Symptom Score (TSS), and Neuropathic Pain Symptoms Inventory (NPSI). ANOVA shows a significantly decreased pain intensity and symptom scores were observed by MNSI, TSS, and NPSI (*P* < 0.0001) during the treatment period and compared to baseline.

**Figure 2:**
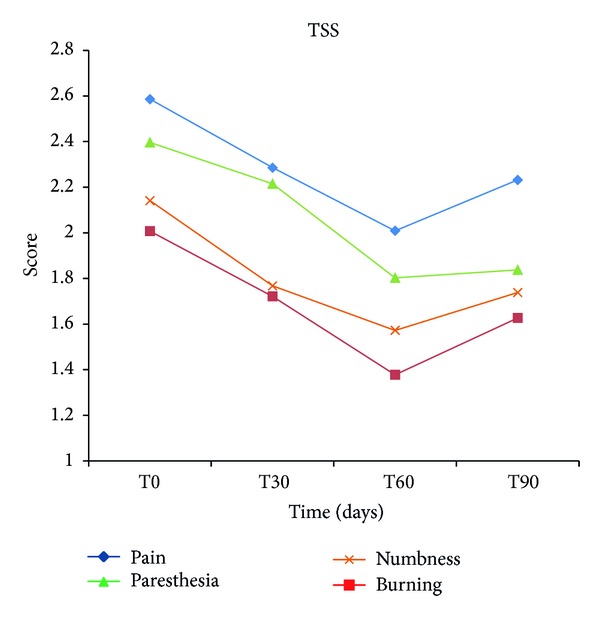
Effect of micronized PEA on each single neuropathic pain symptom assessed by Total Symptom Score (TSS). The intensity and frequency of pain, burning, paresthesia, and numbness, evaluated by TSS, show a significant mitigation (*P* < 0.0001) after 60 days of treatment compared to baseline. This effect persists even one month after treatment discontinuation.

**Figure 3:**
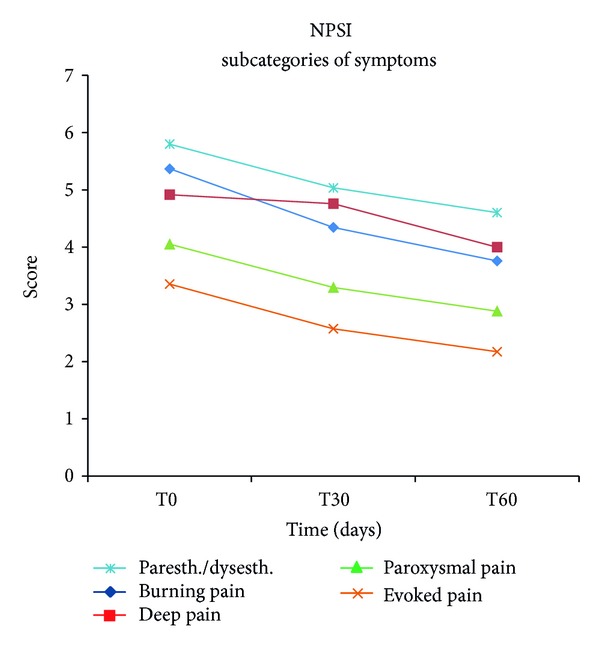
Effects of micronized PEA on single subcategories of neuropathic pain symptoms measured by Neuropathic Pain Symptoms Inventory (NPSI). ANOVA confirmed the significant improvement, compared to baseline (*P* < 0.0001), of the more important characteristics of neuropathic pain: spontaneous superficial pain burning, deep spontaneous pressing pain, paroxysmal pain, and evoked pain (paresthesia/dysesthesia) evaluated by NPSI.

Statistical analysis (ANOVA) of the data demonstrated that treatment with micronized PEA resulted in a significant reduction in the pain symptoms characteristic of diabetic neuropathy already after 30 days. In fact, the median values obtained from MNSI, TSS, and NPSI diminished, compared to baseline (T0), at various observation points until the end of treatment (T60), confirming a significant attenuation (*P* < 0.0001) in the intensity and presence of painful symptoms (Figure 1). The variations in TSS scores were analyzed also separately for each single symptom. The results obtained from this evaluation confirmed that after 60 days of treatment the same significant reduction (*P* < 0.0001) was seen in relation to the intensity and frequency of occurrence in individual symptoms, namely, pain, burning, paresthesia, and numbness (Figure 2). NPSI, which assesses in detail the different categories of symptoms of neuropathic pain, confirmed the significant (*P* < 0.0001) effect of PEA-m in mitigating the intensity of both global neuropathic pain and individual subcategories, such as spontaneous superficial pain, deep spontaneous pain, paroxysmal and evoked pain, and paresthesia/dysesthesia (Figure 3). Moreover, MNSI and TSS carried out one month after cessation of treatment (T90) showed a persistence of the effect achieved (Figures 1 and 2). The Tukey Kramer adjusted test demonstrated no significant difference (*P* > 0.05) between T60 and T90 for every single symptom (Figure 2). Overall assessment of TTS symptoms (Figure 1, Tukey Kramer adjusted test) showed between T60 and T90 a significant increase (*P* < 0.0040) in global mean value which, however, remained significantly lower (*P* < 0.0001) over baseline (T0) and comparable to the value at T30 (*P* > 0.05). Using the McNemar Test, hematology and blood chemistry/urine analyses showed no changes attributable to treatment with micronized PEA, nor were any adverse events noted.

Neuropathic pain results from damage or disease affecting the somatosensory system. Up to 7 to 8% of the Western population is affected and in 5% of persons it may be severe. Diabetes and other metabolic conditions represent the common causes of painful peripheral neuropathies. Neuropathic pain can be very difficult to treat, with less than half of patients achieving partial relief from therapeutics which currently comprise mainly opioids and nonsteroidal anti-inflammatory drugs [39]. The choice of treatment for neuropathic pain should always take into consideration, besides efficacy, safety, and tolerability of the therapy itself, the potential for interaction with other concomitant treatments [40].

There is ever-increasing appreciation for a role of microglia and mast cells in neuropathic pain. As such, pharmacological attenuation of microglial and mast cell activation represents a promising therapeutic avenue for pathologies where neuropathic pain is a major element [41]. We are now aware of the existence of molecules involved in endogenous protective mechanisms activated in the body as a result of different types of tissue damage or stimulation of inflammatory responses and nociceptive fibers. In this context the N-acylethanolamine PEA, which is abundant in the nervous system and produced “*on-demand,*” has seen a remarkable rise in the number of studies published on its anti-inflammatory actions in the past 15 years [13]. A PEA key role may be to maintain cellular homeostasis in the face of external stressors provoking, for example, inflammation. At the same time, there could well be pathological scenarios where PEA endogenous production is inadequate to control the ensuing inflammatory cascade. In such instances, exogenously applied PEA may prove to be beneficial. Indeed, as discussed earlier, a number of preclinical studies show PEA to have anti-inflammatory and analgesic effects in chronic and/or neuropathic pain, as well as in patients suffering from neuropathic pain associated with pathologies of various etiologies [22]. The findings presented in the current study demonstrate that PEA-m treatment resulted in a significant reduction in the pain symptoms characteristic of diabetic neuropathy already after 30 days. The median values obtained from MNSI, TSS, and NPSI diminished, compared to baseline, at various observation points until the end of treatment 60 days later, with no adverse side effects. Limitations of this trial are its open-label nature and small patient population. Notwithstanding these caveats, we believe that our results warrant further investigation in a placebo-controlled, double-blind study.
